# Editorial: Delirium in older persons

**DOI:** 10.3389/fmed.2025.1587624

**Published:** 2025-03-18

**Authors:** Paolo Mazzola, Kevin N. Hascup

**Affiliations:** ^1^Acute Geriatrics Unit, School of Medicine and Surgery, and Fondazione IRCCS San Gerardo dei Tintori, University of Milano-Bicocca, Monza, Italy; ^2^Department of Neurology, Pharmacology, and Medical Microbiology, Immunology and Cell Biology, Dale and Deborah Smith Center for Alzheimer's Research and Treatment, Southern Illinois University School of Medicine, Springfield, IL, United States

**Keywords:** lactate, visfatin, postoperative delirium, elderly, frailty index, anticholinergics, intensive care unit, emergency department

Delirium, a common yet often overlooked condition in the elderly, involves acute confusion and disorientation, often triggered by factors like infections, medications, and chronic illnesses. Delirium is frequently misdiagnosed or dismissed as normal aging, leading to delayed treatment and worse outcomes. Recognition and management are essential for improving quality of life, reducing hospital stays, healthcare costs, and the risk of long-term cognitive decline. This editorial explores biomarkers, medications, and assessment techniques to identify and reduce delirium.

## Serum lactate

Serum lactate levels are vital in managing critically ill patients, especially in predicting intensive care unit (ICU) delirium and mortality. In this retrospective cohort study, “*Associations of serum lactate and lactate clearance with delirium in the early stage of ICU*”, data from the MIMIC-IV database were analyzed to explore the link between lactate levels within 24 h of ICU admission and delirium, as well as lactate clearance rates and 30-day mortality (Qian et al.). Lactic acidosis or hyperlactatemia increased delirium risk, while decreased lactate clearance was associated with higher mortality. These results highlight the need to monitor and manage lactate levels.

## Polypharmacy in Parkinson's disease (PD)

PD presents challenges, especially in older patients with polypharmacy. In the case report, “*Exacerbation of delirium and epileptic seizures in an older man with idiopathic Parkinson's disease due to multiple prescriptions*”, an elderly male with PD and mild renal dysfunction was prescribed 14 medications. He developed delirium and seizures, which improved after reducing his medication load, including amantadine (Yamaguchi et al.). This case highlights the importance of careful drug management in PD, urging clinicians to regularly reassess medications to prevent polypharmacy complications.

## Intraoperative EEG monitoring

Postoperative delirium (POD) is a serious complication that can worsen patient outcomes, increasing morbidity, hospital stays, and long-term cognitive issues. The systematic review, “*Intraoperative electroencephalogram patterns as predictors of postoperative delirium in older patients*”, analyzed 19 studies with 7,229 patients and found that intraoperative EEG patterns, especially burst suppression, increased the risk of POD by 41% (Likhvantsev et al.). This highlights the importance of EEG monitoring during surgery in older patients, offering a potential tool for early detection and prevention strategies to improve outcomes and reduce POD risk.

## Plasma visfatin

Visfatin, a pro-inflammatory cytokine, plays a dual in POD. In the prospective analysis, “*Preoperative plasma visfatin may have a dual effect on the occurrence of postoperative delirium*”, elderly patients scheduled for hip fracture surgery were monitored. Preoperative plasma visfatin levels below 37.87 ng/ml were protective against POD, whereas levels exceeding this threshold increased the risk; an effect mediated by elevated postoperative IL-6 levels (Kang et al.). These findings suggest that preoperative monitoring may help manage POD risk.

## Preoperative anticholinergics

Anticholinergics, used to treat muscle spasms, overactive bladder, and respiratory or gastrointestinal disorders, can increase delirium risk. In the secondary analysis of the randomized, prospective, multicenter study, “*Anticholinergic drug exposure increases the risk of delirium in older patients undergoing elective surgery*”, found that among 899 elderly surgical patients, a higher preoperative anticholinergic burden, measured by the Anticholinergic Risk Scale and Burden Score, was associated with a 2.7-fold increase in POD (Herrmann et al.). The study emphasizes the need to adjust medication regimens before surgery to reduce POD and improve outcomes.

## Leveraging machine learning (ML)

Delirium is a common and serious issue in older ICU patients, leading to poor outcomes. In the study “*Interpretable machine learning model for early prediction of delirium in elderly patients following intensive care unit admission: a derivation and validation study*”, researchers used ML to predict delirium risk in the MIMIC-IV database (Tang et al.). The XGBoost model, enhanced with SHapley Additive exPlanations, offered the most accurate and interpretable predictions. Key predictors included the Glasgow Coma Scale, mechanical ventilation, and sedation. This approach enables personalized risk assessment and could improve early intervention for elderly ICU patients.

## Frailty index (FI) predicts delirium

Frailty, marked by reduced physiological reserve and higher vulnerability, increases the risk of adverse health outcomes like falls and mortality. In the article, “*Frailty index and risk of delirium in hospitalized patients: a two-sample Mendelian randomization study*”, the authors explored the causal link between frailty and delirium (Chen et al.). Using genome-wide data, they found that a higher FI might raise delirium risk. This underscores the need for early frailty assessment in hospitalized patients.

## Emergency department (ED) triage

Delirium is often overlooked in the ED. In the prospective diagnostic study, “*The 4AT scale for rapid detection of delirium in emergency department triage*”, researchers evaluated the 4AT scale for its accuracy and efficiency in triage (Soler-Sanchis et al.). They examined 370 patients aged 65 and older, and found that a score ≥3 had an 85.1% sensitivity and 66.9% specificity in detecting delirium. The scale's use did not extend triage time, making it a valuable tool for early delirium detection without disrupting ED workflow.

## Leveraging caregivers

Early detection of delirium in the ICU is vital for patient outcomes. The Sour Seven Questionnaire adapted into Chinese, was evaluated in the study, “*Translation, cultural debugging, and validation of the Chinese Version of the Sour Seven Questionnaire*” (Zhu et al.). Tested among families of ICU patients in China and compared with the Confusion Assessment Method for ICU, the questionnaire showed high sensitivity (86.3%) and specificity (97.4%). It proved reliable for caregiver screening of ICU delirium, even during online visits, enabling families to assist in early detection.

## Preventing post-stroke delirium

Post-stroke delirium is a serious complication that affects recovery. In the prospective observational study, “*Delirium following mechanical thrombectomy for ischemic stroke – individuals at risk, imaging biomarkers and prognosis*”, 747 patients who underwent mechanical thrombectomy for large vessel occlusion stroke had an 8.2% incidence of delirium, resulting in worse functional outcomes at 90 days. Key risk factors identified included age, sex, anesthesia, infections, stroke etiologies, and medial temporal lobe atrophy scores (Hahn et al.).

In conclusion, addressing delirium in older adults requires a proactive, multidisciplinary approach. Strategies like reducing medications, monitoring biomarkers, and using assessment tools aim to minimize delirium's occurrence and impact. Focusing on personalized care can further reduce delirium risk, improving outcomes and quality of life for elderly patients. Integrating these findings into clinical practice can help reduce the burden of delirium and enhance recovery.

